# Integrating explainable AI and One Health: a new frontier in combating infectious diseases

**DOI:** 10.1016/j.ebiom.2026.106207

**Published:** 2026-03-12

**Authors:** Yanni Cao, Emma Lancaster, Jiyoung Lee, Jianyong Wu

**Affiliations:** a Division of Environmental Health Sciences, College of Public Health, The Ohio State University, Columbus, OH, 43210, USA; b Infectious Diseases Institute, The Ohio State University, Columbus, OH, 43210, USA; c Department of Food Science & Technology, The Ohio State University, Columbus, OH, 43210, USA

**Keywords:** Explainable artificial intelligence (XAI), One Health, Infectious diseases, Predictive modelling

## Abstract

Infectious diseases (IDs) remain a major threat to global health and societal stability. Because most emerging IDs in humans are zoonotic in origin and shaped by environmental contexts, effective prevention and control call for a One Health approach. Machine learning is widely used for ID modelling and forecasting but often lacks interpretability to explain predictions or guide public health action. Explainable AI (XAI) makes complex models interpretable, enabling attribution of predictions and identification of key outbreak drivers. In this Personal View, we argue that embedding XAI within a One Health framework offers a new organising principle for ID intelligence. We highlight emerging applications in surveillance and forecasting, zoonotic spillover, antimicrobial resistance monitoring and optimisation of resource allocation. We also outline key challenges, including data harmonisation, governance, privacy protection and equitable distribution of risks and benefits. Advancing XAI-enabled One Health systems will require collaboration across sectors and methodological innovation.

## Introduction

Infectious diseases (IDs) remain one of the most pressing threats to global health and societal well-being.^1^ The COVID-19 pandemic alone had caused more than 7.1 million reported deaths worldwide as of November 9, 2025.^2^ It also led to the largest global economic contraction since World War II.^3^ These outcomes have highlighted the importance of ID prediction, prevention and more proactive preparedness. Research indicates that over 60% of human IDs are zoonotic, stemming from pathogens transmitted between humans and wild or domestic animals,^4^^,^^5^ while the risk of zoonotic spillover is closely linked to human activities, including habitat destruction.^6^ This underscores the importance of systematically integrating humans, animals, and the environment in ID prevention. In response, the One Health framework has been proposed. The World Health Organization (WHO) defines One Health as ‘an integrated, unifying approach to balance and optimise the health of people, animals, and the environment’.^7^ Essentially, the central concept is that all these three domains are inherently interconnected, with each domain directly or indirectly influencing the others.^8^ In recent years, there has been a growing consensus around the One Health framework for global health and sustainable development.^9^^,^^10^ In the context of IDs, more studies advocate the One Health framework for prevention and control,^11^, ^12^, ^13^ including COVID-19^14^ and bartonellosis.^15^ The value of One Health for the prevention and early detection of disease threats had been recognised globally, leading to its inclusion as an important component of the Global Health Security Agenda.^16^

Against this backdrop, practice-oriented efforts to operationalise One Health for ID surveillance, forecasting, and prevention are accelerating, spanning integrated surveillance platforms that link human, animal, and environmental data streams (e.g., USAID's PREDICT program^17^); systems-thinking frameworks and interdisciplinary systems perspectives^18^; and big-data and AI technologies for multisource data fusion and situational awareness.^19^^,^^20^ In parallel, cross-sector data are expanding in volume, velocity, and variety, with new modalities (e.g., audio and video, mobile app and sensor data) and faster refresh cycles, creating an urgent need for near-real-time data integration and processing. Conventional statistical approaches and black-box machine learning models often fail to provide transparent, decision-oriented rationales. This gap motivates methods that combine performance with interpretability. In this context, explainable artificial intelligence (XAI) offers a powerful tool to enhance understanding, transparency, and trust in complex modelling processes. XAI comprises a growing set of tools that help make machine learning models transparent and interpretable to human users.^21^ Unlike traditional black-box algorithms, XAI methods clarify which factors drive outcomes and why.^22^ Far beyond a technical enhancement, XAI represents a strategic frontier for operationalising One Health in ID contexts, as it makes predictions and responses more interpretable, coordinated, and responsive to real-world complexity.

Recent reviews highlight AI's promise for ID modelling from disease surveillance to epidemic forecasting and spillover prediction, but they largely emphasise accuracy and data integration rather than how model outputs can be made interpretable and actionable for decision-makers across One Health.^23^^,^^24^ We argue that XAI fills this operational gap by translating complex predictions into transparent rationales, enabling shared interpretation, accountability, and coordinated action across sectors. Embedding XAI in One Health goes beyond data integration by enabling mechanism-informed action, shared cross-disciplinary interpretation, and transparent, accountable decision-making.^25^, ^26^, ^27^ In this sense, XAI functions as an implementation layer that operationalizes One Health rather than a technical add-on. In this article, we (i) address that integrating XAI with One Health is essential for combating IDs; then (ii) summarise current applications and illustrative cases of XAI in One Health; thereafter (iii) discuss current challenges and practical considerations; and finally (iv) propose future directions.

## The need for integration

### Complexity of infectious diseases

Despite decades of progress in ID control, a persistent burden of long-standing, emerging, and re-emerging infections remains. For example, tuberculosis continues to pose a major global health threat,^28^ while cholera,^29^ Mpox,^30^ and measles^31^ have re-emerged in multiple regions. Additionally, new human infections of highly pathogenic avian influenza (HPAI)^32^ and dengue virus^33^ are increasingly reported. These recurring crises highlight that IDs are not merely clinical concerns but are deeply intertwined with the stability, resilience, and welfare of societies.^34^^,^^35^ In an era shaped by accelerating globalisation, climate instability, ecological disruption, and social inequity, the landscape of ID threats is becoming increasingly dynamic and difficult to manage.^36^ This underscores the importance of addressing the broader systemic forces that drive disease vulnerability. Rather than stemming from a single cause, IDs arise from a complex web of interacting drivers. These include land-use change, intensified livestock production, global travel and trade, urban crowding, climate variability and vulnerability, inadequate sanitation, antimicrobial resistance (AMR), vector-borne transmission, and zoonotic spillover.^1^^,^^37^^,^^38^ These drivers do not operate in isolation; instead, they intersect and can amplify one another across human, animal, and environmental systems.

### One Health as a foundational framework for infectious disease prediction

Originating in the early 2000s in response to growing concern over pandemic-prone IDs, One Health has since evolved into a coherent conceptual framework for cross-sectoral action.^39^ This framework encourages a collaborative, transdisciplinary strategy that integrates local, national, and global efforts to achieve optimal health for humans, animals, and the environment,^40^ which is critical for preventing, anticipating, and responding to global health threats, as it fosters innovative solutions that address root causes within and across domains.^41^ Using dengue forecasting as an example, one study argues for assessing dengue risk more comprehensively from a One Health perspective.^42^ Here, the animal pillar encompasses vector ecology, the human pillar covers individual and social factors, (e.g., health, socioeconomic status, education, behaviour and demographics), and the environmental pillar includes climatic and geographical factors. This supports cross-sectoral strategies to anticipate, prevent and manage threats across disciplinary and borders. Ultimately, One Health provides a proactive, ecosystem-based approach to interconnected ID risks and long-term health.

### Limitations of existing ID forecasting models

Drawing on prior studies, ID forecasting approaches are commonly grouped into traditional compartmental, statistical, and machine learning models. Traditional compartmental models, such as Susceptible–Infectious–Recovered (SIR) models, have long provided the foundation for ID modelling and remain essential for understanding transmission dynamics because of their transparency and mechanistic clarity.^43^ However, these models often struggle to accommodate the high-dimensional, real-time, and unstructured data sources, and are typically inflexible when additional compartments or pathways are needed.^44^ Besides, the increasing complexity of disease drivers, including climate variables, human mobility, land use, and socio-economic indicators, challenges traditional frameworks that rely on predefined compartments and assumptions. As a result, conventional models may fall short in detecting subtle patterns, adapting to rapidly changing conditions, or capturing cross–sectoral interactions.^45^ Statistical models, such as time-series approaches and regression models, also have limitations, including weak out-of-sample generalisability and poor suitability for multimodal data such as video, mobility, and remote-sensing data.^24^^,^^46^^,^^47^ Generalisability limitations also apply to complex AI models, which may capture highly local associations that do not readily transfer across different One Health settings. Explainability can help identify such context dependence by revealing reliance on local drivers, supporting responsible model adaptation rather than uncritical generalisation.

In response to the growing volume, dimensionality, and heterogeneity of data on ID drivers, alongside stringent timeliness requirements, machine learning methods are increasingly being employed,^46^ and have been widely used to forecast ID outbreaks^45^^,^^48^ as well as anticipate zoonotic spillover events.^49^^,^^50^ Common approaches include supervised learning (e.g., random forests, deep neural networks), semi-supervised learning, unsupervised learning (e.g., clustering and association rule mining), and reinforcement learning.^45^^,^^48^, ^49^, ^50^, ^51^ Despite advances in modelling heterogeneous data, black-box machine learning systems often fail to provide decision-relevant explanations that link diverse predictors to outcomes, limiting scientific understanding and their operational values.

### Importance of XAI

This lack of interpretability in AI models poses serious challenges in One Health, where public health professionals, veterinarians, ecologists, and policymakers must collaboratively respond to emerging threats. In such interdisciplinary contexts, the rationale behind predictions must be clear, accountable, and actionable in the sense that model outputs can be traced and justified in real-world decision making, not just accurate. Evidence from health and public-sector decision-support settings indicates that transparent and explainable models are more likely to foster stakeholder trust, facilitate cross-sector coordination, and support timely and targeted responses.^22^^,^^26^^,^^52^^,^^53^

XAI makes complex models more transparent by identifying key drivers of predictions and how their influence varies across contexts, bridging the gap between predictive performance and operational use. Techniques such as SHapley Additive exPlanations (SHAP) and Local Interpretable Model-Agnostic Explanations (LIME) exemplify how model outputs can be broken down into human-understandable components.^54^ SHAP identifies the most influential contributors to a model's prediction,^25^ while LIME explains the drivers behind individual predictions in specific contexts.^26^ Together, these tools turn opaque algorithms into transparent decision-support systems aligned with the goals of One Health. To illustrate these advantages, we present several use cases of XAI-integrated models and highlight their advantages over conventional statistical models (Table 1).

**Table 1:** Comparative advantages of XAI-integrated models over conventional statistical approaches in recent infectious disease studies.

Disease and research area	Data in One Health framework	Conventional statistical model (Reference)	Optimal machine learning model	XAI approach	Gain from XAI
Dengue, Bangladesh^55^	Human: Poverty. Population density, GDP. Environment: temperature, humidity, rain, wind, vector habitat.	Generalised linear model with the AUC of 0.84	XGBoost with the AUC of 0.89.	Global driver identification with SHAP; Local prediction explanations with LIME.	LIME revealed complex, non-linear thresholds, providing actionable targets that linear coefficients miss.
West Nile Virus, Europe^56^	Animal: *Passeriformes* birds. Vector: *Culex* spp. Abundance. Environment: bioclimatic, Water, etc.	Time-Series/Regression	XGBoost with the AUC ranging from 0.93 to 0.97.	SHAP for Ranking biotic/abiotic drivers and visualising contributions.	SHAP disentangled complex lagged effects.
Malaria, Kenya^57^	Human: Symptoms (Nausea, Fever). Parasite: *Plasmodium* spp. Environment: Rain, temperature.	Linear clinical methods	Random Forest with the accuracy of 98%.	SHAP & DALEX for visualising decision paths for individual patients.	DALEX/SHAP provided instance-level explanations, which mimics clinical reasoning, fostering trust that black box models lack.

GDP: Gross Domestic Product, AUC: Area under the receiver operating characteristic curve.

## Applications of XAI in One Health

### Disease surveillance and prediction

**Fig. 1:**
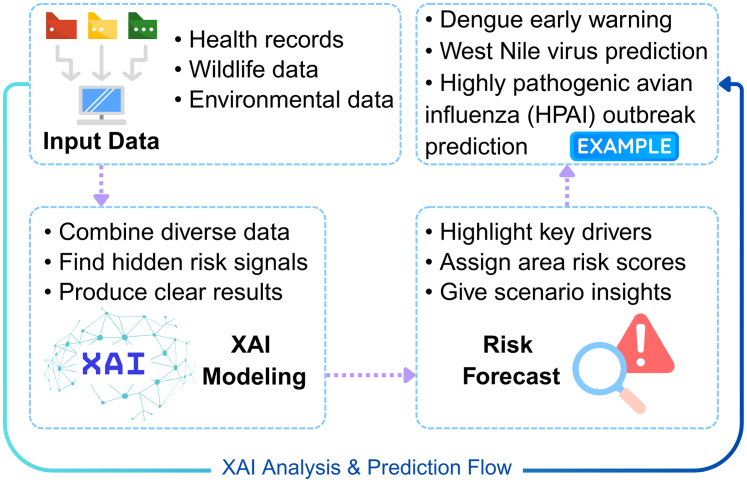
XAI analysis and prediction workflow for multi-source infectious disease surveillance.

Interconnected human, animal, and environmental systems drives ID emergence and spread through complex interactions, such as wildlife movements and human activities.^27^ Growing recognition of these links has underscored the importance of effective cross-sector disease surveillance within One Health frameworks.^17^^,^^58^ In this context, XAI provides a promising approach to addressing the complexity and opacity of disease risk prediction.^47^ By integrating diverse input data, such as health records, wildlife monitoring, and environmental observations, XAI-based models can detect subtle, non-linear ecological precursors, such as specific climatic thresholds or complex vector–habitat interactions, and generate clear, interpretable forecasts.^47^^,^^59^ Incorporating animal health indicators, such as wildlife density or livestock infection rates, further enhances these models’ ability to capture cross-species transmission dynamics central to the One Health framework. Fig. 1 summarises the end-to-end XAI workflow designed for disease surveillance, from integrating multi-source data to producing localised interpretations.

Recent studies illustrate the practical value of this approach. For example, an XAI-enhanced framework in Bangladesh combined climatic, socio-demographic, and land-use data to predict dengue outbreaks and clarify the main factors driving risk.^55^ While this system primarily integrated human and environmental information, SHAP analyses quantified the relative importance of key variables, highlighting the central role of rainfall, population density, and minimum temperature in shaping transmission dynamics. LIME was then used to generate local explanations, showing how these features influenced specific predictions across different regions and time periods. In Europe, a similar XAI approach was applied to West Nile virus prediction.^56^ SHAP values were used to rank the predictors contributing most to outbreak forecasts, identifying preceding-year summer temperatures, seasonal spring temperature anomalies, and *Culex* mosquito abundance as the most influential drivers. Importantly, explainability allowed the model to distinguish the broader eco-climatic factors underlying endemic risk from the specific conditions that triggered the extraordinary 2018 outbreak. Moreover, an XGBoost model interpreted with SHAP was developed to predict HPAI outbreaks at the NUTS-3 level.^60^ The analysis identified poultry density, climatic conditions and environmental indices, together with wild bird distributions, as the main drivers of outbreak risk. These insights informed sentinel surveillance and early warning systems that explicitly incorporate climate patterns, migratory bird ecology and reinfection dynamics, illustrating how XAI can operationalise One Health principles in routine outbreak prediction. Together, these examples demonstrate that XAI can enhance disease surveillance systems by clarifying predictive mechanisms, strengthening actionable interpretation and enabling more responsive, locally informed public health decisions.

### Zoonotic spillover and vector–host–environment interfaces

Unlike population-level ID surveillance, tracking zoonotic spillover requires attention to ecological interfaces where pathogens move between wildlife, livestock, vectors, and humans.^23^ Monitoring these spillover pathways remains a profound challenge because it demands integrating diverse signals from wildlife ecology, livestock management, human behaviours, and environmental change. Subtle shifts, including habitat fragmentation, altered animal movement or microbial contamination of water and soil, often precede zoonotic emergence but frequently go undetected in conventional surveillance systems. In this context, XAI offers a way to transform complex datasets into interpretable risk assessments and actionable insights.

XAI-based models can help detect incipient environmental signals by capturing subtle changes in climate, water quality, soil conditions, wildlife habitats and other environmental factors that precede pathogen spillover. One XAI-based study compared multiple algorithms to predict *Cryptosporidium* and *Giardia* presence in surface water. SHAP showed that low temperatures (<20 °C) and high turbidity drove *Cryptosporidium* contamination, while *Escherichia (E.) coli* counts and turbidity were the main predictors of *Giardia*.^59^ Beyond early detection, these models also excel at predicting localised hotspots and revealing fine-scale spatiotemporal risk patterns that often elude conventional surveillance systems. In Malaysia, a deep neural network combined with LIME was applied to forecast leptospirosis outbreaks, identifying weekly hotspots linked to acidic soils and extensive rubber plantations, and enabling targeted interventions in high-risk communities.^61^

At a broader scale, XAI frameworks are well-suited to quantifying the large-scale drivers of zoonotic spillover and supporting high-resolution decision-making. For example, an integrated machine learning approach linked human modification, livestock density, and cropping intensity to higher spillover risk, while SHAP highlighted crop cover and livestock headcounts as mediators.^62^ In Peninsular Malaysia, human *Plasmodium knowlesi* risk was modelled using an XGBoost ecological niche model with multiple environmental covariates. The model outperformed alternatives, and SHAP highlighted distance to the coastline, elevation, tree cover, historical precipitation, historical tree loss, and distance to forest as dominant predictors, supporting risk mapping for targeted control.^63^ Taken together, these examples illustrate XAI's transformative potential for zoonotic disease tracking from traditional approaches. As One Health surveillance systems evolve, embedding XAI within predictive frameworks offers a unique opportunity to bridge disciplinary divides, strengthen accountability and deliver more precise interventions.

### Monitoring and predicting antimicrobial resistance

Antimicrobial Resistance (AMR) crisis has been recognised as one of the leading global health threats.^64^ While AMR has traditionally been studied in clinical and healthcare settings, increasing research highlights the role of the environment in shaping resistant microbes.^65^ Resistant bacteria and antibiotic resistance genes (ARGs) can evolve, spread, and persist across human, animal, and environmental sectors, making AMR a complex One Health issue.^66^ Although antibiotic resistance is widely monitored in clinical settings, its functions, transmission risks, and broader ecological impacts remain poorly understood, especially in the environment. To address these challenges, XAI can help integrate diverse One Health data to track and explain AMR trends. By combining clinical, prescribing, livestock, and environmental ARG datasets, XAI clarifies cross–domain interactions, explains resistance patterns, and identifies key sources and pathways. XAI can help characterise key antimicrobial mechanisms, including mode-of-action and gene transfer dynamics that link resistance across species and domains. For example, it has been applied to predict antimicrobial mechanisms-of-action from transcriptome data, providing interpretable insights into the features driving resistance patterns and supporting the discovery of novel antibiotics.^67^

The food supply chain is a critical yet under-recognised pathway for AMR transmission, particularly via contamination and cross–sector interactions.^68^ Changes in the supply chain can increase food-safety risks for both humans and animals by increasing exposure to resistant microbes in contaminated food or feed, which may lead to drug-resistant infections. In this context, one study compared SHAP, LIME, and the What-If Tool (WIT) for food fraud and safety evaluation based on speed, explanation scope, and usability.^69^ In light of these emerging applications and the persistent knowledge gaps in AMR surveillance, explainable techniques hold great potential for advancing public health strategies. Accordingly, further exploration of XAI techniques in AMR surveillance is encouraged, as such efforts could enhance public health protection, ensure food safety, and promote sustainable agriculture. Such insights can support more proactive global AMR governance, including surveillance prioritisation and risk communication.

### Optimising intervention strategies and resource allocation

Beyond surveillance and predictive modelling, XAI also plays a critical role in designing proactive strategies. By informing how, where, and why interventions are needed, XAI supports the optimisation of One Health responses across domains. Specifically, it enables interpretable disease surveillance by identifying which variables (e.g., land use, vector density, temperature, precipitation) drive outbreaks and how interactions between human and animal health indicators elevate such risk. This transparency enhances early warning systems that stakeholders can trust and act upon with greater confidence. Guiding intervention strategies is often difficult due to limited resources, including vaccination campaigns, vector control, safe water and sanitation, habitat protection, and wildlife monitoring. To overcome these limitations, particularly in resource-constrained settings, explainability is essential. Given the collaborative nature of One Health, XAI can provide transparent rationales to align decision-making across environmental, animal, and human health sectors. Consequently, XAI can provide effective justification of resource distribution by showing why a particular region, species, or population is prioritised over others. For example, when implementing vaccination campaigns, XAI models can identify high-risk populations and explain why particular communities are prioritised, whether they have higher mobility, recent outbreak history, or low immunity. Such transparency facilitates greater community engagement and public acceptance of interventions.

Unsustainable human practices such as deforestation, urbanisation, water overuse, and air pollution disrupt ecosystems, increase human–animal interactions, and elevate the risk of zoonotic disease transmission.^70^ XAI can promote effective resource management, which are critical for controlling disease spread, ensuring food and water security, and supporting overall well-being across all domains.^71^^,^^72^ One recent study applied a XAI-driven method to investigate the connections among eight universal water quality indicators in Indian river bodies, delivering an interpretable tool for estimation and management.^73^ Another study applied XAI models to uncover how climate extremes and land cover jointly shape wildfire smoke–related PM_2.5_, an emerging contaminant of concern for both human and environmental health.^74^ XAI also allows decision-makers to understand the key factors that drive both our renewable and non-renewable resources, such as air, water, plants, soil, minerals, and fossil fuels. For example, wildlife fisheries can extract the key water quality parameters that are directly influencing fish health and productivity to plan fishing schedules, optimise locations, and reduce the risks of fish mortality.^73^ Additionally, natural ecosystems, such as forests and wetlands, play a crucial role in mitigating climate change, which in turn affects the distribution and severity of many IDs.^72^ XAI-based modelling of natural and managed resources can guide their safe use while helping prevent pathogen contamination. Sustainable management of these resources, in turn, strengthens the resilience of health systems and promotes long-term public and environmental health.

### Meeting stakeholders’ needs

**Fig. 2:**
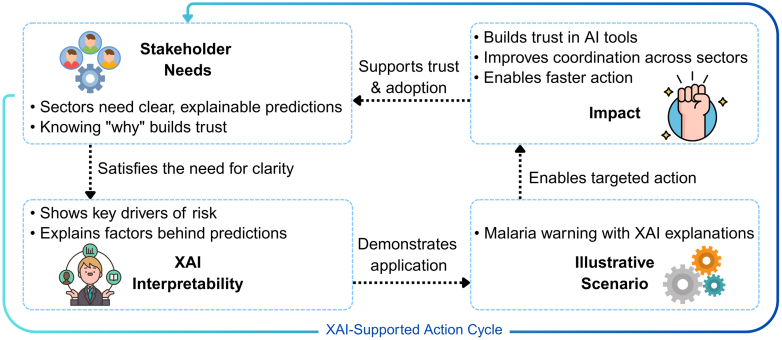
XAI interpretability–action–trust cycle.

Stakeholders across human, animal, and environmental health increasingly view interpretability as essential for deploying AI in practice. In endemic settings, malaria forecasts are of limited value if their rationale is opaque. XAI addresses this by explaining predictions and identifying key risk drivers. For example, an XAI-enhanced malaria model in Kenya used SHAP and descriptive machine learning explanations (DALEX) to pinpoint the factors most strongly influencing malaria risk scores.^57^ These included clinical symptoms like fever and muscle aches, parasite species identification, and environmental variables such as rainfall and temperature. By making these contributions transparent, XAI-enabled models allowed public health teams to determine not only where risk was the highest but also why, supporting more targeted and accountable interventions. This transparency is not merely a technical benefit. It strengthens confidence in AI tools, improves coordination among sectors, and enables faster, evidence-based responses. Over time, this clarity reinforces trust and supports sustained adoption of predictive systems, creating a continuous cycle in which explainability meets stakeholder needs and drives more effective collaboration, as illustrated in Fig. 2.

## Challenges and considerations

XAI plays a critical role not only in surveillance and intervention, but also in building resilience through feedback and adaptation. Its inherent capacity for continual learning allows for iterative improvements in One Health decision-making. Despite these advantages of integrating XAI and One Health in combating IDs, several challenges persist.

A key limitation is that explainability does not inherently imply causal understanding. Many widely used XAI approaches rely on post hoc explanations of complex models, which may be sensitive to data perturbations, modelling choices, or the selection of explanation methods, potentially creating false reassurance if interpreted uncritically in high-stakes health contexts.^52^^,^^53^ These concerns highlight the need to view XAI as a complementary tool rather than a substitute for external validation, domain expertise, and inherently interpretable models. In One Health contexts, especially in highly complex systems, some predictions may arise from non-linear interactions that remain only partially explainable. In these cases, XAI results should be interpreted with biological and ecological plausibility in mind and supported by domain expertise, rather than treated as evidence of mechanistic interactions or causal relationships without further validation.^52^^,^^53^

One of the biggest challenges is integrating heterogeneous data across human, animal, and environmental domains. These datasets often differ in structure, format (e.g., text, imaging, numerical data), spatial and temporal resolution, quality, and accessibility, making standardisation difficult. Such fragmentation constrains the potential of XAI models by reducing interpretability. To support transparent and robust outputs, developing data harmonisation protocols, intersectoral data infrastructure and multimodal XAI frameworks are essential.

Alongside technical challenges, ethical and privacy concerns arise when deploying AI in cross-sectoral health contexts. Sensitive information from medical records, zoonotic surveillance systems, and environmental monitoring must be protected. Because the nature of One Health is interdisciplinary, managing data governance, privacy, and compliance with government regulations can be challenging. Explainability serves as an ethical safeguard by exposing biases and unintended consequences in model reasoning. Biases in data and model outputs can lead to the stigmatisation of communities, unequal health interventions, and underrepresented wildlife populations. Thus, ethical considerations must be taken to ensure accountability and fairness across animal, human, and environmental health. While explainability methods such as SHAP and LIME enhance transparency at model level, system-level transparency is equally vital for real-world adoption. This broader dimension includes clear communication of model outputs, rationale for interventions, data governance, and mechanisms for public accountability.

Despite growing interest and promising applications, XAI remains a developing field that requires further refinement. Most existing explainability methods were initially designed for general-purpose, non-spatial tasks and are not optimised for the complex, multi-scale data environments typical of One Health applications. However, recent studies have shown that XAI-enhanced models can capture spatial patterns in ways comparable to conventional approaches.^75^ These applications, however, require further validation across longitudinal and cross-sectoral contexts, particularly given the trade-off between model interpretability and predictive performance.

## Future directions

Unlocking XAI's potential in One Health demands coordinated investment and integration; otherwise, it will remain underused. Here, “investment” includes interoperable data infrastructure, analytic capacity, workforce training, decision-support tools, and governance frameworks for privacy, fairness, and auditability. Many stakeholders, including local public health practitioners, veterinary officers, and policy analysts, lack training or resources to interpret model outputs. Educational programs and dashboards tailored to user needs can help democratise access to XAI tools.

Deeper interdisciplinary collaboration is essential. Algorithmic performance alone is insufficient without integrating expertise from epidemiology, veterinary science, ecology and environmental health with data and computer science. AI developers should co-design models with One Health practitioners to reflect real-world constraints and priorities. Robust policy and governance frameworks are also essential to ensure that XAI is deployed ethically and equitably. National and global agencies should develop guidelines that incorporate responsible AI principles, such as transparency, fairness, and accountability, into One Health systems. These standards should include validation procedures, reporting requirements, and equity-focused assessments.

Finally, future research must address persistent technical gaps. These include the need for scalable XAI methods that can handle spatiotemporal data, support domain adaptation, and function across complex, multimodal datasets. Key priorities include enhancing explainability for graph neural networks, real-time surveillance models, and multi-level causal frameworks. As these innovations mature, they can shift XAI from retrospective interpretation toward prospective guidance, improving timeliness and precision in cross-sectoral responses. Moving forward, efforts must be collaborative, ethically grounded, and driven by both scientific innovation and operational feasibility, ensuring that XAI becomes a reliable and trusted tool for ID surveillance, forecasting, and management across species and systems.

## Conclusion

Integrating XAI into the One Health framework offers a transformative opportunity to improve the prediction, prevention, preparedness, and control of IDs. By making complex models interpretable, XAI enables targeted interventions and supports cross-sector decision-making in dynamic systems. As illustrated here, XAI not only enhances technical transparency but also promotes trust and equity, empowering stakeholders across human, animal, plant, and environmental domains to act on shared health insights. Realising this potential will require sustained efforts to embed transparency in AI systems, strengthen interdisciplinary collaboration, and advance innovation in explainability methods adapted to One Health contexts. We call on researchers, practitioners, and policymakers to jointly pursue ethical, effective, and scalable applications of XAI that support resilient, equitable, and sustainable health outcomes across species and sectors.

## Outstanding questions

A key outstanding question is how XAI can be integrated into One Health to enable coordinated decision-making across human, animal, and environmental systems. Related questions include how XAI can be operationalised in real-time cross-sector surveillance, what data streams are required to build reliable and explainable models that generalise across domains, and how XAI can support causal reasoning rather than correlation-driven interpretation in ID modelling.

**Search strategy and selection criteria**

We searched literature from PubMed and Google Scholar up to January 25, 2026. Queries combined One Health and infectious disease concepts (e.g., “One Health,” “infectious diseases,” “zoonotic,” “spillover,” and “forecasting/surveillance”) with AI and explainability-related terms (e.g., “explainable artificial intelligence (XAI),” “model interpretability,” SHAP, and LIME). Terms related to common modelling approaches (e.g., compartmental and statistical/time-series models), AMR/ARG terminology, and disease-specific keywords (e.g., influenza/H5N1 and dengue) were added as needed. Searches used Boolean operators (e.g., AND/OR) where appropriate. We included peer-reviewed English-language articles, prioritising studies from the past five years. Conference abstracts, meeting reports, and non–peer-reviewed supplements were excluded; authoritative sources (e.g., WHO) were cited when needed. Only a small number of peer-reviewed conference papers were included when they were relevant. References were selected based on relevance and the quality.

## Contributors

YC and JW conceptualised the study. YC, EL and JW wrote the original draft. YC, EL, JL and JW reviewed and edited the manuscript. All the authors have read and approved the final manuscript.

## Declaration of interests

We declare no competing interests.
